# Anthropometric Features and Third-Fourth Degree Perineal Tears

**DOI:** 10.3390/jpm13030545

**Published:** 2023-03-18

**Authors:** Arrigo Fruscalzo, Alice Novak, Camilla Somma, Anjeza Xholli, Virginia Michelerio, Federico Prefumo, Ambrogio P. Londero, Angelo Cagnacci

**Affiliations:** 1 Clinic of Obstetrics and Gynecology, University Hospital of Fribourg, 1752 Fribourg, Switzerland; 2 Clinic of Obstetrics and Gynecology, DAME, Academic Hospital of Udine, 33100 Udine, Italy; 3 Academic Unit of Obstetrics and Gynecology, IRCCS Ospedale San Martino, 16132 Genoa, Italy; 4 Obstetrics and Gynecology Unit, IRCCS Istituto Giannina Gaslini, 16147 Genova, Italy; 5 Department of Neuroscience, Rehabilitation, Ophthalmology, Genetics, Maternal and Infant Health, University of Genoa, 16132 Genova, Italy

**Keywords:** third- and fourth-degree perineal tears, obstetrical complications, vaginal delivery, anal sphincter laceration, feto-maternal body-mass index

## Abstract

The main objective of this study was to evaluate the association between maternal and fetal anthropometric characteristics and third- and fourth-degree perineal tears. This retrospective cohort study considered all consecutive pregnancies from 2011 to 2017 at a single Institution. The inclusion criteria were: singletons who delivered vaginally during the study period, the presence of information on maternal pre-pregnancy weight, maternal height, and weight of the newborn. The feto-maternal body-mass index (BMI) was calculated as neonatal weight in kg on maternal height in squared meters (kg/m^2^). In total, 5397 singleton-term pregnancies were included; the prevalence of third-fourth-degree perineal tears was 0.47%. The most predictive factors were: nulliparity, feto-maternal BMI, neonatal weight, gestational age at delivery, and neonatal head circumference. After adjustment in multivariate analysis, the only independent predictors were nulliparity and fetomaternal BMI. The AUC of the final multivariate model was 73.54% (95% CI 65.65–81.42). Furthermore, feto-maternal BMI and gestational age had a significant direct correlation. Nulliparity and feto-maternal BMI are the two best predictors for third and fourth-degree perineal tears in our setting. Confirming this association in future research and integrating it into a decision algorithm on delivery timing could reduce obstetric damage to the anal sphincter.

## 1. Introduction

Perineal tears are injuries of the perineum related to childbirth involving the perineal soft tissues and muscles. They are defined as high-degree perineal tears if the anal sphincter complex is also concerned and are classified as third- and fourth-degree according to whether the anal epithelium is involved. High-degree perineal tears are not only major contributors to short-term morbidity due to local pain and wound breakdown but are also linked to subsequent chronic pelvic pain and dyspareunia, as well as a large spectrum of symptoms of loss of bowel control, including anal incontinence [1]. A recent meta-analysis estimated the risk of third- and fourth-degree perineal laceration at 5.7% [2]. However, the incidence can vary considerably according to the obstetric population considered [3,4,5].

In a previous study in our population, we observed an incidence of 1.3% of third- and fourth-degree perineal tears in a cohort of women who gave birth vaginally and were questioned about their quality of life one year after delivery [6]. This prevalence was much higher in women with symptoms of pelvic floor disorders (3.33%) or with dyspareunia (4.62%) [7,8]. All this highlights how third and fourth-degree perineal tears have important implications on quality of life and women’s health beyond the period immediately following childbirth. These long-time sequelae are demanding tools to predict their onset, which might be helpful in childbirth planning and timing. Knowing the risk factors associated with high-degree perineal tears may assist health providers in developing new strategies for reducing their occurrence and practitioners in adapting them to the local setting. The most common risk factors include increasing maternal age, race and ethnicity, nulliparity, operative vaginal delivery, episiotomy, prolonged second stage, fetal occiput posterior presentation, and large fetal weight [9,10]. In particular, this latter seems to play a role even if different cut-offs have been proposed [11,12]. This could be explained by the different types of populations considered, including their maternal height.

In order to overcome this limitation, researchers argued that the feto-maternal body-mass index (BMI), the ratio between neonatal weight and maternal height, could be a good index for predicting obstetric damage to the anal sphincter. This new parameter was proposed for the first time at the International Urogynecological Association (IUGA) world congress in Toronto in 2010 as an ultrasound index for predicting the risk of damage to the anal sphincter during childbirth [13]. However, to our knowledge, only one peer-reviewed study was recently conducted confirming this hypothesis and the association with other unfavorable outcomes such as non-elective cesarean delivery and instrumental deliveries [14]. The main objective of this study was to evaluate the association between maternal and fetal anthropometric characteristics, particularly the feto-maternal BMI and third- or fourth-degree perineal tears.

## 2. Materials and Methods

This retrospective cohort study considered all consecutive deliveries from 2011 to 2017 at the Obstetrics and Gynecology Unit of “Santa Maria Della Misericordia” University Hospital in Udine, Italy. The inclusion criteria were: singletons who delivered vaginally during the study period; the presence of information on maternal pre-pregnancy weight, maternal height, and weight of the newborn. Conversely, the exclusion criteria were childbirth by cesarean section, twin pregnancies, and the absence of neonatal and maternal anthropometric information.

Maternal-fetal and neonatal data were routinely collected from the clinical database. The data considered were the following: maternal age, parity, maternal height, pre-pregnancy maternal weight, pre-pregnancy BMI, assisted reproductive techniques, and geographic origin. The pregnancy outcomes considered were: gestational age at delivery, mode of labor onset, delivery mode, presence of third- and fourth-degree perineal tears, hypertensive disorders of pregnancy (HDP), gestational age at birth, neonatal length, neonatal head circumference, placental weight, neonatal sex, neonatal weight, Apgar score at the 1st and 5th minutes, infant small for gestational age (SGA), large for gestational age (LGA), presence of neonatal congenital anomalies, neonatal resuscitation, and admission to neonatal intensive care unit (NICU). The feto-maternal BMI was calculated as neonatal weight in kg on maternal height in squared meters (kg/m^2^).

Gestational age was calculated from the last known menstrual period and confirmed by ultrasound examination during the first and second trimesters of pregnancy. Hypertension was defined as a systolic blood pressure greater than or equal to 140 mmHg or a diastolic blood pressure greater than or equal to 90 mmHg [15,16]. We considered hypertensive disorders of pregnancy: pre-eclampsia, eclampsia, gestational hypertension, and pre-eclampsia superimposed on chronic hypertension [17,18].

Pre-eclampsia was defined as hypertension in combination with proteinuria [15,16]. Proteinuria was defined as urinary excretion of 0.3 g of protein or greater in 24 h (this usually correlates with 30 mg/dL or greater in a random urine determination). Gestational hypertension was similarly defined as pre-eclampsia but without proteinuria, and eclampsia was defined as pre-eclampsia but with seizures [15,16]. Chronic hypertension was defined as hypertension present before the 20th week of gestation [15,16]. This study defined SGA as a neonatal weight below the 3rd or 10th centile, and LGA as a neonatal weight above the 90th or 97th centile [19,20]. Preterm delivery was considered before 37 weeks of gestational age.

Our hospital protocol concerning perineal laceration broadly overlaps with the recommendations of the Royal College of Obstetricians and Gynaecologists (RCOG) for the prevention of obstetric anal sphincter injuries (OASIS) [10]. Experienced midwives assisted all deliveries with medical staff present for eventual complications. The episiotomy procedure was always performed by midwives based on their subjective clinical judgment. The episiotomy incision was performed mediolaterally as previously described [6]. Episiotomy was only achieved when circumstances dictated the shortening of the second stage of labor, such as maternal exhaustion, non-reassuring fetal heart rate, the need for vacuum instrumentation, shoulder dystocia, and when a severe perineal tear was judged to be imminent. Additionally, the birth attendants always used maneuvers to protect the perineum during the second stage of labor. The diagnosis of OASIS was made at delivery by clinical examination of experienced medical staff. A third-degree tear was defined as a tear extending into the anal sphincter, and a tear extending further into the lining of the anus or rectum was considered a fourth-degree tear. Our local policy recommends a cesarean section when a patient has a prior history of anal sphincter injury.

### Data Analysis

Statistical analysis was performed using the R program (version 4.2.2; R Core Team (2022)). R: A language and environment for statistical computing. R Foundation for Statistical Computing, Vienna, Austria. URL https://www.R-project.org/) [21]. Differences with *p* < 0.05 were considered significant. Data are presented as the median and interquartile range (IQR) for non-parametric continuous variables; mean ± standard deviation in case of continuous parametric variables. Dichotomous variables are presented as percentage and absolute values, excluding missing values (NA). The results of the logistic regression models are presented as odds ratios (OR) and 95% confidence intervals (CI). The Kolmogorov–Smirnov test tested continuous variables’ distribution to establish the distribution’s normality. The following statistical tests were also used in the case of continuous variables: *t*-test for parametric variables and Wilcoxon test for non-parametric variables. Where appropriate, the Chi-square test or Fisher’s exact test was used for dichotomous variables. Finally, a logistic regression analysis was performed considering third- and fourth-degree lacerations as the dependent variable and possible risk factors as independent variables. The multivariate model evaluated all potential predictive factors with a *p* < 0.100 in the univariate analysis. All the variables and their interactions were entered into the initial multivariate model. In cases where the interactions were not significant, the analysis of the model without interaction was performed. The accuracy of the predictive models was assessed using the area under the curve (AUC) of the receiver operating characteristic (ROC) curves. Furthermore, the differences between the AUCs were evaluated by Delong’s test.

## 3. Results

**Figure 1:**
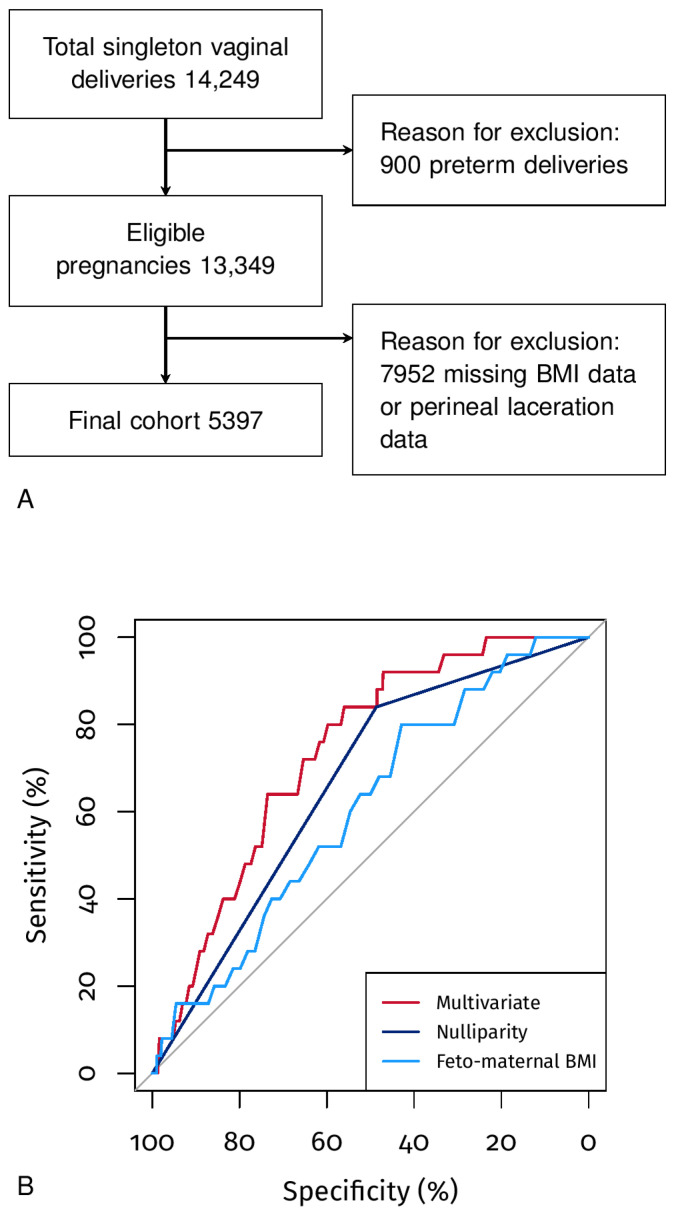
Panel (**A**), Flowchart of the study. Panel (**B**), ROC curves of the univariate and multivariate models.

In total, 5397 out of 13,349 deliveries of singleton pregnancies at term performed vaginally complied with the inclusion criteria (Figure 1A). The prevalence of third- or fourth-degree perineal tears was 0.46%.

Table 1 shows the characteristics of the population analyzed; the median age was 32 years (IQR 28–35) and 51.49% of the women were nulliparous. The median feto-maternal BMI was 1.23 kg/m^2^ (IQR 1.12–1.34) and had a directly proportional and significant correlation with the gestational age at delivery (rho = 0.32, *p* < 0.05). Table 1 also shows the characteristics of the newborns, 49.32% of which were male and had an average weight of 3375 g (IQR 3110–3664).

Table 2 shows the differences between the controls and women with a third- or fourth-degree perineal laceration. It should be noted that these women were more often nulliparous (*p* < 0.05), had a higher feto-maternal BMI (*p* < 0.05), and had a higher gestational age at delivery (*p* = 0.071). Table 2 also shows the differences in neonatal characteristics between controls and third- or fourth-degree perineal lacerations. Among pregnancies with a third or fourth-degree perineal tear, we observed a larger neonatal weight (*p* = 0.072) and a larger head circumference (*p* = 0.107). As expected, there was no association between SGA and third- or fourth-degree perineal injuries.

**Figure 2:**
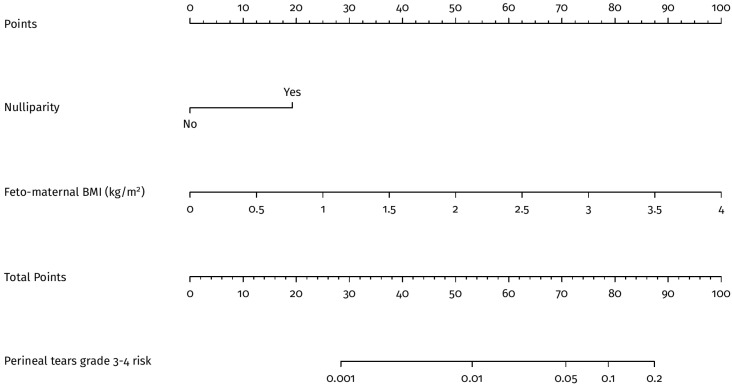
Nomogram of the multivaraite model.

Univariate and multivariate logistic regressions were also performed, considering the most significant variables. In particular, the most predictive characteristics after a stepwise selection of the variables were found to be nulliparity (AUC 66.33%, 95% CI 58.97–73.69) and feto-maternal BMI (AUC 60.72%, 95% CI 50.64–70.81) (Table 3). The multivariate model that considers nulliparity and feto-maternal BMI presents an AUC of 73.54% (95% CI 65.65–81.42) (Figure 1B). In particular, we observed differences between the multivariate model AUC and the following univariate AUCs: nulliparity (*p* < 0.05), feto-maternal BMI (*p* < 0.05), gestational age at delivery (*p* = 0.079), neonatal weight (*p* < 0.05), or neonatal head circumference (*p* < 0.05). Figure 2 also shows the nomogram of the multivariate model, which indicates how the feto-maternal BMI plays an essential role in risk stratification. In particular, a nulliparous woman 1.56 m tall with a child weighing 3900 g has a risk of 1 to 5%, i.e., much higher than the 0.46% of the general population; the same woman with a child weighing 2900 g has a risk of less than 1%. In both of the above examples, if the woman is parous, the risk is below 1% (Figure 2).

## 4. Discussion

This retrospective study showed that feto-maternal BMI is significantly and independently associated with third- and fourth-degree perineal tears.

In recent years, the feto-maternal BMI has emerged as a potential ultrasound index for predicting the risk of damage to the anal sphincter during childbirth [1]. However, despite the initial interest in this index, more research has yet to be conducted to confirm its utility. A recent peer-reviewed study has provided evidence supporting the association between feto-maternal BMI and adverse outcomes during childbirth, including vaginal instrumental and non-elective cesarean delivery deliveries [2]. Our study builds on this research by corroborating the correlation between feto-maternal BMI and birth lacerations of the anal sphincter. Taken together, these findings suggest that the feto-maternal BMI may have important clinical implications for predicting and managing complications during childbirth. This data assumes particular importance if we consider that LGA fetuses do not seem to be a predictive factor for third- and fourth-degree perineal tears in our population. At the same time, the relationship between maternal (height) and fetal (birth weight) anthropometric characteristics show a significant predictivity.

According to the literature, duration of second-stage labor, operative vacuum delivery, prior history of anal sphincter injury, maternal age, gestational age at delivery, and maternal race/ethnicity were associated with increased risk of injury to the anal sphincter [10,22]. In our study, in addition to nulliparity, we only confirm gestational age at delivery and neonatal weight as risk factors. It should be noted that our retrospective series lacks data on the duration of the second stage of labor and a previous history of anal sphincter injury. In the case of a previous history of anal sphincter injury, a delivery by elective cesarean section was indicated in our local setting. Considering the ultrasound fetal weight estimate and the ultrasound estimate of the fetal head circumference, a recent study highlighted how these parameters seem not to have a significant predictive contribution to obstetric damage of the anal sphincter (significant results in univariate but not in multivariate analysis) [23]. These results are similar to our findings, where both neonatal weight and neonatal head circumference are significantly associated with third- and fourth-degree perineal tears, but both are less predictive than feto-maternal BMI.

As highlighted in previous studies conducted in our obstetric population, third- and fourth-degree perineal lacerations with damage to the anal sphincter appear to be associated with major pelvic floor disorders even after the index delivery [6,7,8]. A recent study confirming this finding shows that damage to the anal sphincter during childbirth has a substantial impact on the affected women [24]. In this study, more than half of the women assessed during the follow-up reported symptoms associated with anal sphincter damage [24]. Furthermore, almost half reported that this impacted future birth choices [24]. From this data, it can be argued that there is a long-term impact on the health care system attributable to anal sphincter damage from childbirth and that a better prediction and prevention of these adverse outcomes could be a helpful strategy in reducing the long-term impact of anal sphincter damage [24]. Possible alternative management is to consider programming the timing of induction at a gestational age in which the feto-maternal BMI in nulliparous patients reflects an acceptable risk of high-degree perineal lacerations. This strategy is supported by the observation that the feto-maternal BMI increases with the increase in gestational age and that the gestational age at delivery of patients with third- and fourth-degree perineal lacerations is closer to 40 weeks than to 39 weeks of gestation. Therefore, a planned induction in these patients just after 39 weeks of gestation could reduce high-degree perineal lacerations. Interestingly, a planned induction at 39 weeks of gestation in nulliparous women has been shown to be protective against other major adverse obstetric outcomes [25,26].

### Strengths and Weaknesses of the Study

This study thoroughly evaluates the feto-maternal BMI as a potential risk factor for high-degree perineal lacerations in a large cohort of women. However, the study’s retrospective design and a large amount of missing data weaken the strength of the results. Additionally, the study did not evaluate several known parameters associated with perineal tears, which further limits the reliability of the results. Our clinical policy recommends an elective cesarean section for women with a history of anal sphincter injury during previous childbirth, excluding multiparous women with a higher risk of experiencing vaginal lacerations during vaginal delivery. However, our results align with previous literature [10], and it is unlikely that our cesarean policy had significantly influenced the increased risk observed in the nulliparous group. Moreover, the study was conducted at a single center, which may limit the generalizability of the findings to other populations with different baseline characteristics. Therefore, future studies, ideally large multicenter prospective trials, should be conducted to confirm the results of this study. In light of these limitations, further research is needed to fully understand the potential utility of the feto-maternal BMI index as a predictor of perineal lacerations during childbirth and to identify effective strategies for mitigating its associated risk.

## 5. Conclusions

Nulliparity and feto-maternal BMI are the two best predictors of third- and fourth-degree perineal lacerations in our setting. Integrating these risk factors into a decision algorithm for delivery management could potentially reduce obstetric damage to the anal sphincter.

## Figures and Tables

**Table 1:** Population description.

Patient Characteristics	
Maternal age (years)	32 (28–35)
Nulliparity	51.49% (2779/5397)
Mother height (cm)	165 (161–170)
Maternal pre-pregnancy BMI (kg/m^2^)	22 (20–24)
Feto-maternal BMI (kg/m^2^)	1.23 (1.12–1.34)
Gestational age at delivery (weeks)	39 (39–40)
Macro-region of origin	
West Europe/Italy	73% (3937/5393)
East Europe	16.08% (867/5393)
Sub-Saharan Africa	3.8% (205/5393)
Arabian countries	3.49% (188/5393)
Asia	2.48% (134/5393)
Other	1.15% (62/5393)
HDP	1.45% (76/5240)
Conception mode	
Spontaneous	98.55% (5319/5397)
Ovulation induction/IUI	0.43% (23/5397)
IVF/ICSI	1.02% (55/5397)
Labor-induced/augmented	28.66% (1504/5248)
Childbirth mode	
Spontaneous delivery	89.09% (4808/5397)
Operative vaginal delivery	10.91% (589/5397)
Neonatal characteristics	
Neonatal male sex	49.32% (2662/5397)
1st minute Apgar score	9 (8–9)
5th minute Apgar score	9 (9–9)
Neonatal weight (grams)	3375 (3110–3664)
Neonatal weight (MoM)	1 (1–1)
Placental weight (grams)	580 (510–655)
Neonatal length (cm)	50 (49–51)
Neonatal head circumference (mm)	345 (335–352)
SGA (<3rd centile)	1.74% (94/5397)
SGA (<10th centile)	7.93% (428/5397)
LGA (>90th centile)	12.47% (673/5397)
LGA (>97th centile)	4.98% (269/5397)
Neonatal CPR	1.22% (66/5397)
NICU hospitalization	1.04% (56/5397)

**Table 2:** Comparison between pregnancies with and without grade 3–4 perineal tears.

	Controls (5372)	Perineal Tears Grade 3–4 (25)	*p*
Patient characteristics			
Maternal age (years)	31.54 (±5.40)	31.20 (±5.80)	0.774
Nulliparity	51.34% (2758/5372)	84.00% (21/25)	<0.05
Maternal pre-pregnancy BMI (kg/m^2^)	22.64 (±3.89)	21.96 (±2.91)	0.254
Feto-maternal BMI (kg/m^2^)	1.23 (±0.17)	1.29 (±0.15)	<0.05
Conception mode			
Spontaneous	98.57% (5295/5372)	96.00% (24/25)	0.283
Ovulation induction/IUI	0.43% (23/5372)	0.00% (0/25)	0.743
IVF/ICSI	1.01% (54/5372)	4.00% (1/25)	0.137
Gestational age at delivery (weeks)	39.00 (39.00–40.00)	40.00 (39.00–41.00)	0.071
Macro-region of origin			
West Europe/Italy	73.03% (3921/5369)	66.67% (16/24)	0.492
East Europe	16.07% (863/5369)	16.67% (4/24)	1.000
Sub-Saharan Africa	3.78% (203/5369)	8.33% (2/24)	0.231
Arabian countries	3.48% (187/5369)	4.17% (1/24)	0.574
Asia	2.48% (133/5369)	4.17% (1/24)	0.454
Other	1.15% (62/5369)	0.00% (0/24)	1.000
HDP	1.41% (76/5372)	0.00% (0/25)	1.000
Labor induction/augmentation	28.61% (1495/5225)	39.13% (9/23)	0.266
Vaginal operative delivery	10.87% (584/5372)	20.00% (5/25)	0.144
Neonatal characteristics			
Neonatal male sex	49.26% (2646/5372)	64.00% (16/25)	0.141
1st minute Apgar score	9.00 (8.00–9.00)	9.00 (8.00–9.00)	0.658
5th minute Apgar score	9.00 (9.00–9.00)	9.00 (9.00–10.00)	0.387
Neonatal weight (grams)	3375.00 (3106.00–3664.25)	3515.00 (3380.00–3650.00)	0.072
Neonatal weight (MoM)	1.01 (0.93–1.09)	1.03 (0.98–1.07)	0.383
Placental weight (grams)	580.00 (510.00–655.00)	600.00 (535.00–641.00)	0.642
Neonatal length (cm)	50.00 (49.00–51.00)	50.00 (50.00–51.00)	0.539
Neonatal head circumference (mm)	345.00 (335.00–352.00)	349.00 (343.00–350.00)	0.107
SGA (<3rd centile)	1.75% (94/5372)	0.00% (0/25)	1.000
SGA (<10th centile)	7.97% (428/5372)	0.00% (0/25)	0.258
LGA (>90th centile)	12.49% (671/5372)	8.00% (2/25)	0.761
LGA (>97th centile)	4.97% (267/5372)	8.00% (2/25)	0.356
Neonatal CPR	1.21% (65/5372)	4.00% (1/25)	0.265
NICU hospitalization	1.02% (55/5372)	4.00% (1/25)	0.230

**Table 3:** Analysis by logistic regression, dependent variable presence of grade 3–4 perineal tears.

	OR (CI.95)	*p*	AUC (CI.95)
Nulliparity	4.98 (1.71–14.52)	<0.05	66.33% (58.97–73.69)
Sub-Saharan Africa	2.31 (0.54–9.91)	0.258	52.28% (46.62–57.93)
Mother height (cm)	0.98 (0.92–1.05)	0.618	46.49% (35.31–57.67)
Pre-pregnancy BMI (kg/m^2^)	0.95 (0.84–1.07)	0.380	53.57% (42.83–64.31)
Pre-pregnancy weight (kg)	0.98 (0.94–1.02)	0.279	55.59% (45.88–65.31)
Feto-maternal BMI (kg/m^2^)	5.37 (1.09–26.42)	<0.05	60.72% (50.64–70.81)
Neonatal weight (grams)	1.00 (1.00–1.00)	0.092	60.42% (51.73–69.11)
LGA (>97th centile)	1.77 (0.41–7.58)	0.443	51.79% (45.90–57.69)
Gestational age at delivery (weeks)	1.39 (0.96–2.03)	0.084	60.12% (48.23–72.00)
Neonatal head circumference (mm)	1.02 (1.00–1.04)	0.106	59.31% (49.54–69.07)
Vaginal operative delivery	2.05 (0.77–5.48)	0.153	54.56% (46.55–62.58)
Multivariate model (*)			73.54% (65.65–81.42)
Nulliparity	6.05 (2.00–18.35)	<0.05	
Feto-maternal BMI (kg/m^2^)	10.36 (2.12–50.6)	<0.05	

(*) Multivariate model selected using a stepwise procedure. The differences are as follows between the predictivity of the multivariate model, respectively: nulliparity *p* < 0.05, feto-maternal BMI *p* < 0.05, gestational age at delivery *p* = 0.079, neonatal weight *p* < 0.05, neonatal head circumference *p* < 0.05.

## Data Availability

The data that support the findings of this study are available, but restrictions apply to the availability of these data, which were used under license for the current study, and so are not publicly available. Data are however available from the authors upon reasonable request and with permission of the Internal Review Board.
